# Interleukin-6 and thyroid-stimulating hormone index predict plaque stability in carotid artery stenosis: analyses by lasso-logistic regression

**DOI:** 10.3389/fcvm.2024.1484273

**Published:** 2024-12-09

**Authors:** Li Zhigao, Qin Jiabo, Zheng Lei, Qiao Tong

**Affiliations:** ^1^ Department of Vascular Surgery, Nanjing Drum Tower Hospital Clinical College of Nanjing Medical University, Nanjing, China; ^2^ Department of General Surgery, Nanjing Drum Tower Hospital Clinical College of Nanjing Medical University, Nanjing, China; ^3^ Department of Vascular Surgery, The Affiliated Drum Tower Hospital of Nanjing University Medical School, Nanjing, China

**Keywords:** carotid artery stenosis, thyroid hormone sensitivity, stability of plaques, lasso-logistic regression, nomogram

## Abstract

**Objective:**

To develop and validate a new prediction model based on the Lass-logistic regression with inflammatory serologic markers for the assessment of carotid plaque stability, providing clinicians with a reliable tool for risk stratification and decision-making in the management of carotid artery disease.

**Methods:**

In this study, we retrospectively collected the data of the patients who underwent carotid endarterectomy (CEA) from 2019 to 2023 in Nanjing Drum Tower Hospital. Demographic characteristics, vascular risk factors, and the results of preoperative serum biochemistry were measured and collected. The risk factors for vulnerable carotid plaque were analyzed. A Lasso-logistic regression prediction model was developed and compared with traditional logistic regression models. The Akaike information criterion (AIC) and Bayesian information criterion (BIC) were used to evaluate the performance of three models.

**Results:**

A total of 131 patients were collected in this study, including 66 (50.4%) in the vulnerable plaque group and 65 (49.6%) in the stable plaque group. The final Lasso-logistic regression model included 4 features:IL-6, TSH, TSHI, and TT4RI; AIC = 161.6376, BIC = 176.0136, both lower than the all-variable logistic regression model (AIC = 181.0881, BIC = 261.5936), and the BIC was smaller than the stepwise logistic regression model (AIC = 154.024, BIC = 179.9007). Finally, the prediction model was constructed based on the variables screened by the Lasso regression, and the model had favorable discrimination and calibration.

**Conclusions:**

The noninvasive prediction model based on IL-6 and TSHI is a quantitative tool for predicting vulnerable carotid plaques. It has high diagnostic efficacy and is worth popularizing and applying.

## 1 Introduction

Carotid artery stenosis is a primary risk factor for ischemic stroke, a condition responsible for a large proportion of all stroke cases. This stenosis primarily results from atherosclerosis, where narrowing of the vessel lumen due to atherosclerotic plaques is the main characteristic (1). Clinically, carotid artery stenosis often presents with cerebral ischemia and stroke, contributing to a growing global stroke burden as stroke incidence shifts toward younger populations (2). Consequently, the evaluation and treatment of carotid artery disease have been central topics in cardiovascular and neurological research.

While stenosis severity has traditionally guided risk assessment, research indicates that the characteristics of carotid plaques—such as volume, composition, and stability—are critical in determining stroke risk. Indeed, carotid stenosis alone may not fully predict stroke occurrence (3). Thus, identifying vulnerable, instability-prone plaques is crucial for stroke prevention and for assessing cardiovascular event risk (4). Identifying and managing these plaques can aid in clinical decision-making, especially for patients undergoing carotid endarterectomy (CEA), where plaque stability impacts postoperative outcomes and risks (5). Vulnerable plaques, marked by inflammation and intraplaque neovascularization, have been associated with adverse events post-surgery, including thrombosis, restenosis, and even stroke (6–8).

Traditional imaging methods help assess plaque size and location but have limitations in predicting rupture risk (9, 10). Given that vulnerable plaques exhibit distinct histopathologic features—such as lipid-rich necrotic cores, a fragile fibrous cap, and inflammatory activity (11–13)—biomarker-based assessments are increasingly considered as adjunctive tools (14). Research into blood-based biomarkers, including high-sensitivity C-reactive protein and interleukins, has shown associations with cardiovascular events and may help in assessing plaque stability (15–17). Thyroid hormones have been suggested to influence atherosclerotic plaque stability through mechanisms involving lipid metabolism, inflammation, oxidative stress, and endothelial function, yet their role remains under debate. Indicators such as TSH and FT4, reflecting overall thyroid hormone levels, have been linked to an increased risk of atherosclerosis, particularly when TSH is high or FT4 is low (18, 19). However, given the complexity of the hypothalamic-pituitary-thyroid axis, single serum markers may not capture thyroid function adequately. Composite thyroid hormone sensitivity indices offer a more comprehensive approach to understanding the relationship between thyroid function and carotid atherosclerotic plaque stability, though their specific relevance to plaque vulnerability requires further study.

Considering the potential collinearity among thyroid hormone indices, lipid metabolism markers, and serum inflammation indicators, we chose to use Lasso regression for variable selection and model development. This approach helps mitigate multicollinearity, allowing for the identification of the most relevant predictors and reducing overfitting risk.

Therefore, our study aims to develop and validate a clinical predictive model centered on serologic markers for assessing carotid plaque stability. We envision this model as a non-invasive, cost-effective adjunct to existing imaging-based diagnostic approaches, providing clinicians with a practical risk stratification tool for managing carotid artery disease.

## 2 Materials and methods

### 2.1 Patients

This single-center retrospective study consisted of patients from the Department of Vascular Surgery in Nanjing Drum Tower Hospital between 2019 and 2023 who underwent carotid endarterectomy. Inclusion criteria were as follows: Age ≥45 years, with no gender restriction. Diagnosis of carotid stenosis confirmed by carotid ultrasound examination within the past 3 months. Underwent carotid endarterectomy at our hospital and isolated carotid plaques were obtained. Informed about the study details, and signed the informed consent form. Exclusion criteria were the presence of hematologic systemic diseases or malignant tumors. Presence of other cardiovascular diseases (e.g., severe coronary artery disease, valvular heart disease, atrial fibrillation). History of abnormal thyroid function or thyroid surgery. Use of thyroid hormone replacement therapy or antithyroid medications, such as methimazole or propylthiouracil. Presence of chronic kidney disease, hepatic, or other organ insufficiency. Presence of autoimmune diseases (e.g., Sjögren's syndrome, Takayasu arteritis, ulcerative colitis, Crohn's disease). Patients with incomplete e s sential clinical data, such as preoperative age and relevant laboratory indicators, were excluded from the study. General information of the patients and preoperative examination results were collected for this study, which was approved by the Ethics Committee of Nanjing Drum Tower Hospital (No. 2024-866-01).

### 2.2 Detection methods and observational indexes

#### 2.2.1 Basic data

Gender, age, body mass index (BMI), history of hypertension, diabetes mellitus, and stroke were documented for each study subject upon hospitalization.

#### 2.2.2 Laboratory indices

Routine preoperative serum biochemistry data were collected upon hospitalization, including albumin, glucose, triglycerides, total cholesterol, C-reactive protein, hemoglobin, procalcitonin, IL-6, D-dimer, thyroid hormones, and others.

#### 2.2.3 Plaque stability assessment

Histological characteristics of vulnerable plaques include active inflammation, large necrotic cores, thin fibrous caps, and intraplaque hemorrhage (11–13). Macrophages not only initiate inflammation in ruptured plaques but also degrade the extracellular matrix through protease secretion, leading to fibrous cap thinning and fracturing. Reduced smooth muscle cells diminish extracellular matrix synthesis and collagen repair, resulting in decreased fibrous cap thickness. Vulnerable plaques were assessed using a cumulative score based on intraplaque macrophages, collagen fibers, contractile smooth muscle cells, and intraplaque hemorrhage (20). Plaque processing involved: immunohistochemistry for CD68 and α-SMA to label macrophages and smooth muscle cells, respectively, quantified using ImageJ; Masson staining to detect collagen fiber content, also quantified with ImageJ; Prussian blue staining to evaluate intraplaque hemorrhage. Specific scoring criteria included: macrophage infiltration (none/slight = 0 points, moderate/severe = 1 point), collagen fiber quantity (moderate/majority = 0 points, none/small amount = 1 point), smooth muscle cell presence (moderate/majority = 0 points, none/small amount = 1 point), and intraplaque hemorrhage (absent = 0 points, present = 1 point). Higher scores indicate poorer plaque stability, with scores ≤2 classified as stable plaques and >2 as vulnerable plaques (Supplementary Figure S1).

#### 2.2.4 Calculation of thyroid hormone sensitivity-related indices

Serum concentrations of TSH, FT3, and FT4 were measured using a fully automated immunochemiluminescence kit. The reference ranges for TSH, FT3, and FT4 were 0.27–4.2 mIU/L, 3.1–6.8 pmol/L, and 12–22 pmol/L. Indices reflecting central thyroid hormone sensitivity include TSHI, TT4RI, and TFQI. TSHI is a simple index proposed by Jostel et al. for assessing pituitary sensitivity to thyroid hormones. Maximum pituitary TSH reserve is assessed by extrapolating the TSH feedback inhibition from the measured FT4 concentration to the standardized uninhibited TSH, assuming the FT4 value to be 0 (21, 22). The TT4RI index, first proposed by Yagi et al., assesses pituitary sensitivity to thyroid hormones (23). TFQI, a quartile-based thyroid feedback index, was introduced by Laclaustra's team. It quantifies the deviation of the pituitary's response to the inhibitory effects of thyroid hormones in a continuous manner, showing the deviation of TSH measurements from actual values. This index is advantageous as it remains relatively stable even in the presence of abnormal thyroid function (24, 25). TSHI, TT4RI, and TFQI reflect central sensitivity to thyroid hormones, with higher values indicating lower central sensitivity. The calculated values based on existing studies are presented in Supplementary Table S2.

#### 2.2.5 Lasso-logistic modeling fundamentals

In this study, we employ the Least Absolute Shrinkage and Selection Operator (Lasso) regression method. The Lasso method falls under the umbrella of coefficient regularization techniques, leveraging penalization within the context of least squares regression (26). Let (Xi, yi), i = 1, …, *n* represent the observed values from *n* independent samples, where Xi = (Xi.1, …, Xi, j) denotes the jth attribute value of the ith individual, and yi ∈ {0, 1} indicates the outcome categories, with 0 denoting a stable carotid plaque and 1 denoting a vulnerable carotid plaque. The coefficient estimation is expressed as follows:

$$\begin{array}{rl} {\hat{\beta }}^{\mathrm{LASSO}} & =\arg\underset{\beta }{\min}\overset{n}{\underset{i=1}{\sum }}\{y_{i}(\beta_{0}+\overset{J}{\underset{\;j=1}{\sum }}x_{i,j}\beta_{j})\\ & \;-\log[1+\exp(\beta_{0}+\overset{J}{\underset{\;j=1}{\sum }}x_{i,j}\beta_{j})]\}+\lambda \overset{J}{\underset{\;j=1}{\sum }}|\beta_{j}| \end{array}$$

where ${\hat{\beta }}^{\mathrm{LASSO}}$ represents the coefficient estimate of the Lasso-logistic regression model. *λ* serves as the regularization parameter, controlling the impact of the penalty term through its adjustment. The choice of *λ* is critical, influencing the extent of compression; larger values of *λ* lead to increased sparsity in parameter estimation, reducing non-zero parameter independent variables and enhancing variable selection within the model. The most commonly used method for *λ* estimation is 10-fold cross-validation, which selects the *λ* associated with the smallest error as the optimal tuning parameter.

#### 2.2.6 Model fitting evaluation

The most fundamental method of evaluating model performance involves the direct calculation of metrics such as average absolute error, average variance, coefficient of determination (*R*²), and adjusted coefficient of determination (*R*²_adj), among others, selecting the model with the smallest error and highest correlation. Dichotomous classification models are typically evaluated using Receiver Operating Characteristic (ROC) curves and the Area Under the Curve (AUC), where a larger AUC indicates better classification performance. However, these evaluation methods share a common limitation in that they only assess model performance, irrespective of model complexity. In contrast, information criteria adeptly balance model complexity and performance, scoring models using probabilistic statistics.

##### 2.2.6.1 The Akaike information criterion (AIC)

AIC assesses the goodness of fit of a statistical model, based on entropy, which not only considers the complexity of the estimated model but also evaluates the model's fit to the data (27). Generally, a larger sample size results in a smaller AIC value, indicating better model fit. Therefore, models with the smallest AIC value are preferred during the fitting process.

##### 2.2.6.2 The Bayesian information criterion (BIC)

BIC developed on Bayesian probability principles, aims to estimate partially unknown states using subjective probabilities and correct occurrence probabilities using Bayesian formulas (28). It then makes optimal decisions based on expected values and corrected probabilities. Similar to AIC, BIC maximizes likelihood function estimation, with smaller BIC values indicating better model fit. In contrast to BIC, AIC imposes a smaller penalty on model parameters and tends to select more complex models, while BIC imposes stricter penalties and favors simpler models with fewer parameters.

Both AIC and BIC have strengths and weaknesses in evaluating model effects; therefore, this study combines both to comprehensively assess model fitting. To compare variable selection using the Lasso method, this study applies traditional logistic regression, stepwise logistic regression, and Lasso-logistic regression models, evaluating their fitting effects using AIC and BIC.

#### 2.2.7 Statistical analyses

All statistical analyses were performed using the R-4.3.0 software. Quantitative data that conformed to normal distribution were expressed as Mean ± SD and independent samples *t*-test was used for comparison between groups; quantitative data that did not conform to normal distribution were expressed as M (P25, P75), and Mann-Whitney *U*-test was used for comparison between groups; and categorical data were expressed as the number of cases (percentage), and Pearson's chi-square test was used for comparison between groups. A Lasso-logistic regression model was developed using the “glmnet” software package to explore the factors associated with vulnerable plaques, and the selection of the reconciliation parameter *λ* was performed using cross-validation, which was compared with all-variable logistic regression (univariate followed by multivariate, with multifactorial included at *p* < 0.2) and stepwise logistic regression (backward, conditional, *α* in = 0.05, *α* out = 0.10). Akaike information criterion (AIC) and Bayesian information criterion (BIC) were used to evaluate the fit goodness of the model. The receiver operating characteristic curve (ROC) curve was used to analyze the predictive value of relevant clinical variables on carotid plaque stability. The area under the curve (AUC) and calibration curve were used to assess the differentiation and accuracy of the prediction model. Nomograms of the Lasso-logistic regression model and calibration curves were plotted using the “rms” package. The test level was taken as *α* = 0.05 (two-side).

## 3 Results

### 3.1 Baseline data of enrolled patients

**Figure 1:**
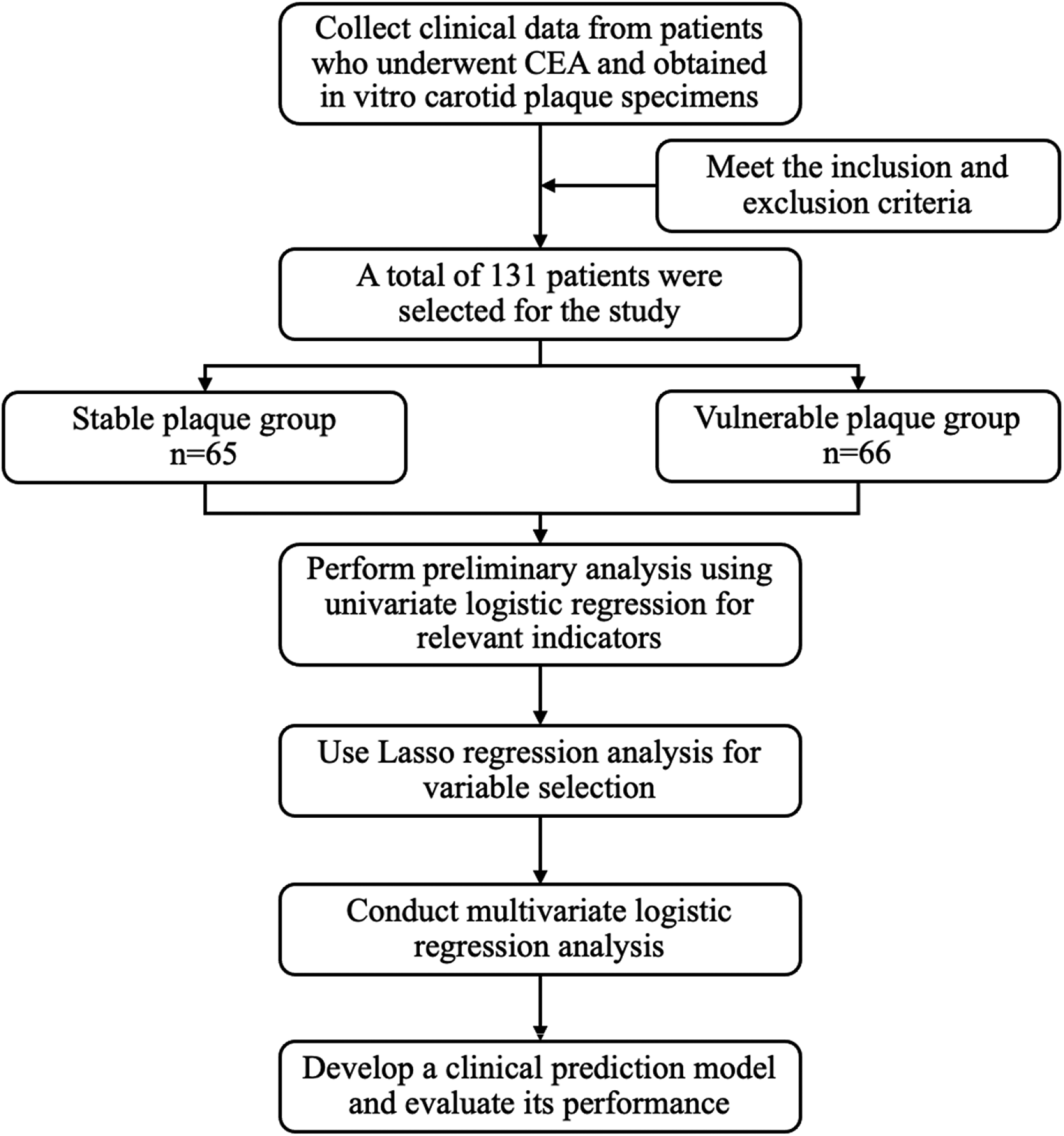
Flow chart of study design.

A total of 131 patients who underwent carotid endarterectomy were included in this study, including 107 (81.68%) males and 24 (18.32%) females, as illustrated in Figure 1. Their average age was (67.61 ± 9.16) years. According to the plaque vulnerability score, 66 cases (50.38%) were vulnerable plaques and 65 cases (49.62%) were stable plaques (Supplementary Table S3). Comparing the vulnerable plaque group with the stable plaque group, there were no statistically significant differences in gender, age, BMI, smoking, history of hypertension, history of diabetes, history of stroke, albumin, glucose, triglycerides, total cholesterol, H-cholesterol, L-cholesterol, Apo AI, Apo B, C-reactive protein, hemoglobin, procalcitonin, D-dimer, FT3, and FT4 (*p* > 0.05). There was a statistical difference between the two groups in terms of alcohol consumption history. Compared with patients in the carotid plaque stabilization group, patients in the plaque vulnerability group had higher IL-6, TSH, TSHI, TT4RI, and TFQI, and the difference was statistically significant (*P* < 0.05) (Table 1).

**Table 1:** Baseline data of 131 patients.

Features	Total (*n* = 131)	Stable (*n* = 65)	Vulnerable (*n* = 66)	Statistics	*P*-value
Gender (*n*, %)				*χ*² = 0.89	0.345
Female	24 (18.32)	14 (21.54)	10 (15.15)		
Male	107 (81.68)	51 (78.46)	56 (84.85)		
Age (years)	67.61 ± 9.16	66.17 ± 8.92	69.03 ± 9.23	*t* = −1.80	0.074
BMI (kg/m^2^)	23.98 ± 3.22	24.18 ± 3.03	23.78 ± 3.40	*t* = 0.71	0.480
Smoking history (*n*, %)				χ² = 0.08	0.775
No	79 (60.31)	40 (61.54)	39 (59.09)		
Yes	52 (39.69)	25 (38.46)	27 (40.91)		
Alcohol history (*n*, %)				χ² = 3.95	**0** **.** **047**
No	104 (79.39)	47 (72.31)	57 (86.36)		
Yes	27 (20.61)	18 (27.69)	9 (13.64)		
Hypertension (*n*, %)				χ² = 0.06	0.801
No	33 (25.19)	17 (26.15)	16 (24.24)		
Yes	98 (74.81)	48 (73.85)	50 (75.76)		
Type 2 diabetes (*n*, %)				χ² = 0.46	0.498
No	87 (66.41)	45 (69.23)	42 (63.64)		
Yes	44 (33.59)	20 (30.77)	24 (36.36)		
Stroke (*n*, %)				χ² = 2.75	0.097
No	63 (48.09)	36 (55.38)	27 (40.91)		
Yes	68 (51.91)	29 (44.62)	39 (59.09)		
Alb (g/L)	39.70 (38.50, 41.20)	39.70 (39.20, 41.30)	39.60 (38.02, 41.08)	Z = −1.08	0.279
Glu (mmol/L)	4.99 (4.42, 5.85)	5.08 (4.60, 5.87)	4.80 (4.34, 5.71)	Z = −1.40	0.162
TG (mmol/L)	1.17 (0.81, 1.67)	1.23 (0.84, 1.68)	1.15 (0.78, 1.58)	Z = −0.87	0.384
TC (mmol/L)	3.69 (3.17, 4.22)	3.72 (3.19, 4.22)	3.65 (3.04, 4.21)	Z = −0.24	0.813
HDL (mmol/L)	1.10 (0.90, 1.33)	1.04 (0.88, 1.32)	1.15 (0.97, 1.35)	Z = −1.04	0.297
LDL (mmol/L)	1.90 (1.60, 2.54)	1.93 (1.62, 2.41)	1.88 (1.54, 2.63)	Z = −0.12	0.903
Apo AⅠ (g/L)	1.07 (0.93, 1.22)	1.05 (0.93, 1.22)	1.10 (0.92, 1.23)	Z = −0.50	0.616
Apo B (g/L)	0.62 (0.56, 0.79)	0.62 (0.57, 0.77)	0.62 (0.54, 0.79)	Z = −0.16	0.876
CRP (mg/L)	3.60 (2.50, 5.45)	3.60 (2.70, 5.20)	3.50 (2.50, 5.70)	Z = −0.03	0.978
Hb (g/L)	134.00 (124.00,145.50)	134.00 (122.00, 145.00)	134.00 (124.25, 145.75)	Z = −0.30	0.763
PCT (ng/ml)	0.05 (0.03, 0.17)	0.04 (0.03, 0.13)	0.05 (0.03, 0.19)	Z = −1.21	0.227
IL-6 (pg/ml)	6.90 (3.60, 15.32)	5.70 (2.41, 13.65)	9.30 (4.41, 15.96)	Z = −2.23	**0**.**026**
D-dimer (mg/L)	0.33 (0.21, 0.58)	0.31 (0.18, 0.51)	0.35 (0.25, 0.63)	Z = −1.11	0.266
TSH (mIU/L)	2.25 (1.53, 3.42)	1.74 (1.38, 2.40)	2.91 (2.04, 3.63)	Z = −4.24	**<**.**001**
FT3 (pmol/L)	4.64 (4.28, 4.92)	4.64 (4.41, 4.92)	4.62 (4.21, 4.97)	Z = −0.62	0.533
FT4 (pmol/L)	16.10 (14.75, 17.60)	16.30 (15.00, 17.50)	15.90 (14.07, 17.95)	Z = −0.24	0.813
TSHI	2.95 (2.65, 3.33)	2.75 (2.39, 3.03)	3.20 (2.85, 3.45)	Z = −4.49	**<**.**001**
TT4RI	35.36 (25.38, 52.61)	28.39 (23.43, 39.02)	45.33 (33.68, 56.30)	Z = −4.52	**<**.**001**
TFQI	0.02 (−0.21, 0.24)	−0.09 (−0.27, 0.05)	0.12 (−0.11, 0.35)	Z = −3.45	**<**.**001**

Abbreviations are provided in Supplementary Materials.

The bold values indicate *P* < 0.05.

### 3.2 Factors associated with plaque stability: univariate logistic regression analysis

Univariate analysis revealed that the vulnerable plaque group had higher levels of TSH, TSHI, TT4RI, and TFQI. The ORs for these variables were as follows: TSH, OR = 2.05 (95% CI: 1.44–2.91); TSHI, OR = 4.41 (95% CI: 2.05–9.48); TT4RI, OR = 1.05 (95% CI: 1.03–1.07); and TFQI, OR = 6.57 (95% CI: 2.10–20.57). All associations were statistically significant, with *P*-values < 0.05 (Table 2).

**Table 2:** Univariate logistic regression analysis of factors associated with plaque stability in patients with carotid artery stenosis.

Features	*Β*	S.E	Z	*P*	OR (95% CI)
Gender (*n*, %)					
Female					1.00 (Reference)
Male	0.43	0.46	0.94	0.347	1.54 (0.63–3.77)
Age (years)	0.04	0.02	1.78	0.076	1.04 (1.00–1.08)
BMI (kg/m^2^)	−0.04	0.05	−0.71	0.477	0.96 (0.86–1.07)
Smoking history (*n*, %)					
No					1.00 (Reference)
Yes	0.10	0.36	0.29	0.775	1.11 (0.55–2.23)
Alcohol history (*n*, %)					
No					1.00 (Reference)
Yes	−0.89	0.45	−1.95	0.051	0.41 (0.17–1.00)
Hypertension (*n*, %)					
No					1.00 (Reference)
Yes	0.10	0.40	0.25	0.801	1.11 (0.50–2.44)
Type 2 diabetes (*n*, %)					
No					1.00 (Reference)
Yes	0.25	0.37	0.68	0.498	1.29 (0.62–2.66)
Stroke (*n*, %)					
No					1.00 (Reference)
Yes	0.58	0.35	1.65	0.099	1.79 (0.90–3.58)
Alb (g/L)	−0.08	0.07	−1.15	0.252	0.93 (0.81–1.06)
Glu (mmol/L)	0.06	0.09	0.67	0.502	1.07 (0.89–1.28)
TG (mmol/L)	−0.15	0.22	−0.68	0.499	0.86 (0.56–1.33)
TC (mmol/L)	0.08	0.16	0.51	0.610	1.09 (0.79–1.50)
HDL (mmol/L)	0.36	0.58	0.62	0.535	1.43 (0.46–4.43)
LDL (mmol/L)	0.13	0.19	0.66	0.512	1.14 (0.78–1.66)
Apo AⅠ (g/L)	0.10	0.80	0.13	0.897	1.11 (0.23–5.32)
Apo B (g/L)	0.63	0.65	0.98	0.328	1.88 (0.53–6.71)
CRP (mg/L)	0.04	0.03	1.41	0.158	1.04 (0.99–1.10)
Hb (g/L)	0.00	0.01	0.39	0.694	1.00 (0.98–1.03)
PCT (ng/ml)	1.03	0.93	1.11	0.268	2.80 (0.45–17.36)
IL-6 (pg/ml)	0.03	0.02	1.77	0.077	1.03 (1.00–1.06)
D-dimer (mg/L)	0.32	0.29	1.07	0.283	1.37 (0.77–2.44)
TSH (mIU/L)	0.72	0.18	4.01	**<** **.** **001**	2.05 (1.44–2.91)
FT3 (pmol/L)	−0.10	0.29	−0.36	0.717	0.90 (0.51–1.58)
FT4 (pmol/L)	−0.02	0.08	−0.21	0.835	0.98 (0.85–1.14)
TSHI	1.48	0.39	3.79	**<**.**001**	4.41 (2.05–9.48)
TT4RI	0.05	0.01	4.06	**<**.**001**	1.05 (1.03–1.07)
TFQI	1.88	0.58	3.23	**0**.**001**	6.57 (2.10–20.57)

B, beta coefficient; S.E, standard error of the mean; CI, confidence interval; OR, odds ratio; CI, confidence interval.

### 3.3 Variable selection and results in multivariate logistic regression

All-variable logistic regression and stepwise logistic regression (backward, conditional, *α* in = 0.05, *α* out = 0.10) were established. The stability of plaque served as dependent variable. Statistically different correlations in the univariate analysis served as the independent variables; Lasso-logistic regression was established utilizing the relevant factors selected by Lasso regression.

#### 3.3.1 Correlation analysis and multiple covariance diagnosis among independent variables

**Figure 2:**
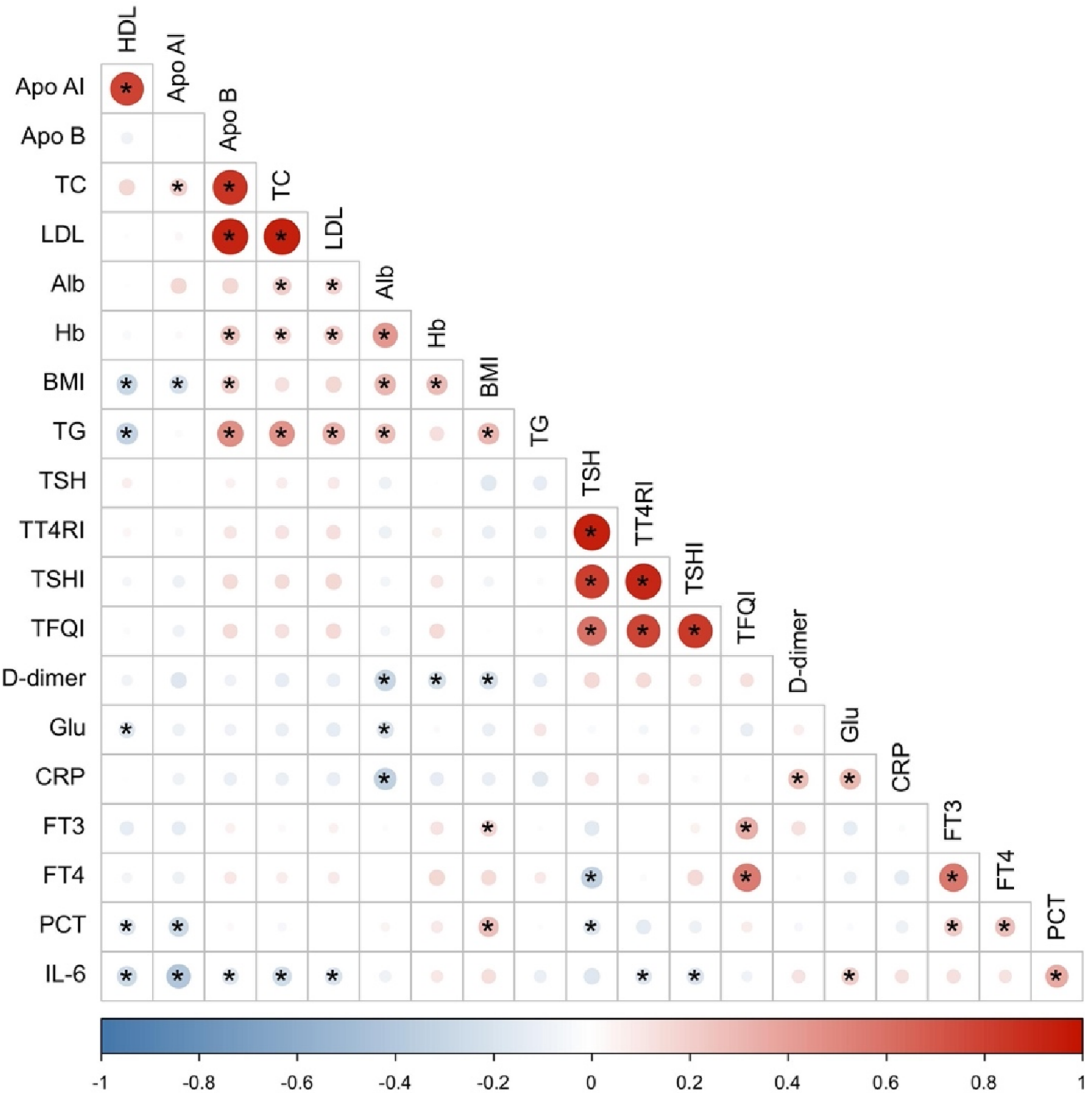
Heat map of correlation between quantitative independent variables. *Denotes statistically significant correlations with *p* < 0.05.

Considering the correlation among different independent variables, the Pearson correlation coefficient (29) was chosen to analyze the correlation among all quantitative independent variables, and finally, a total of 6 items (LDL, Apo B, Apo AI, TSHI, TT4RI, TFQI) showed covariance problems. (Figure 2) Multiple covariance diagnosis was performed for all variables using variance inflation factor as well as tolerance. If VIF value >10 and tolerance <0.1 were used as criteria for covariance (30), a total of 9 items (TC, HDL, LDL, Apo B, TSH, FT4, TSHI, TT4RI, TFQI) showed severe covariance (Supplementary Table S4).

#### 3.3.2 Results of lasso regression analysis

**Figure 3:**
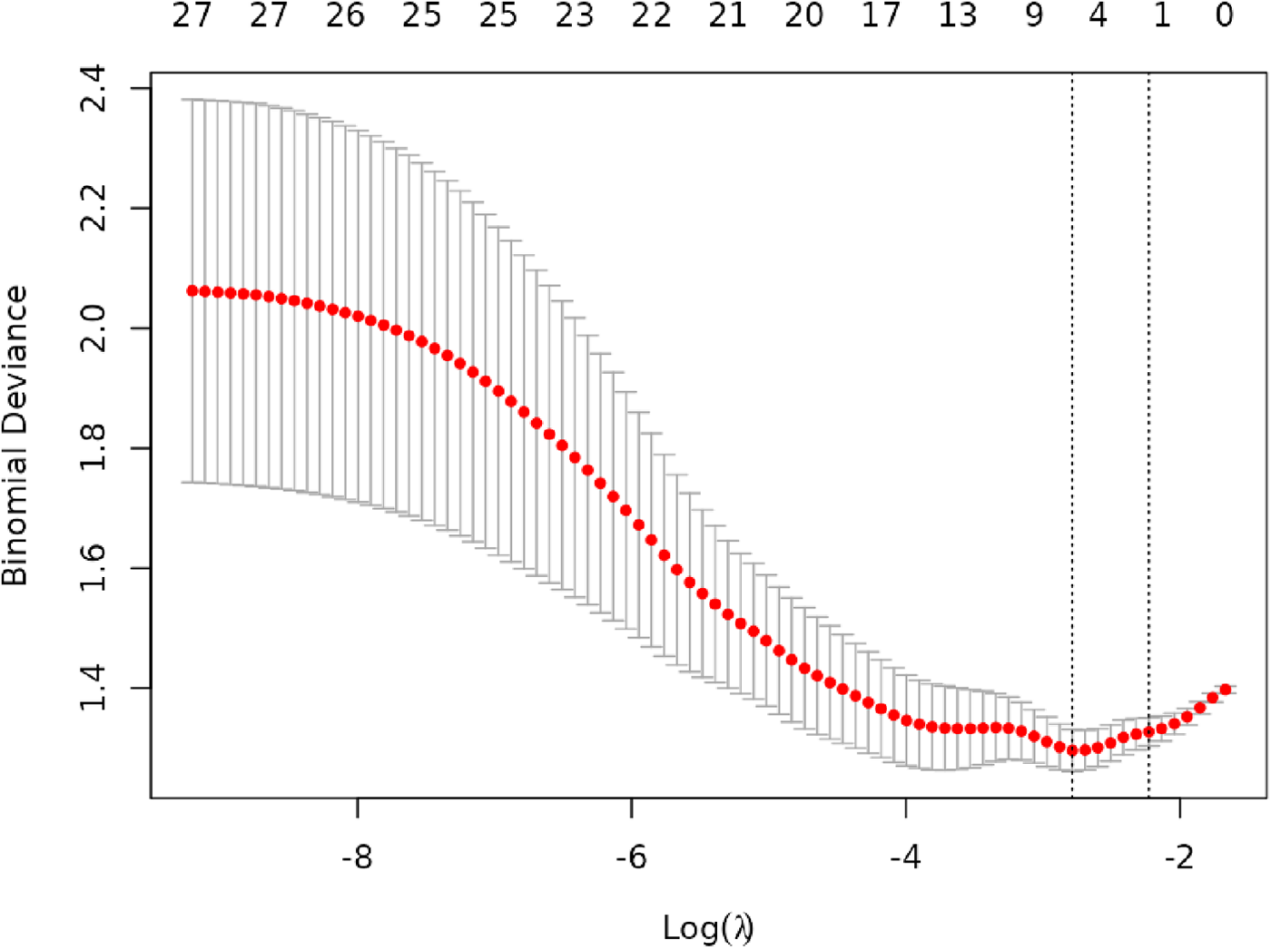
Cross-validation plot of lasso regression.

**Figure 4:**
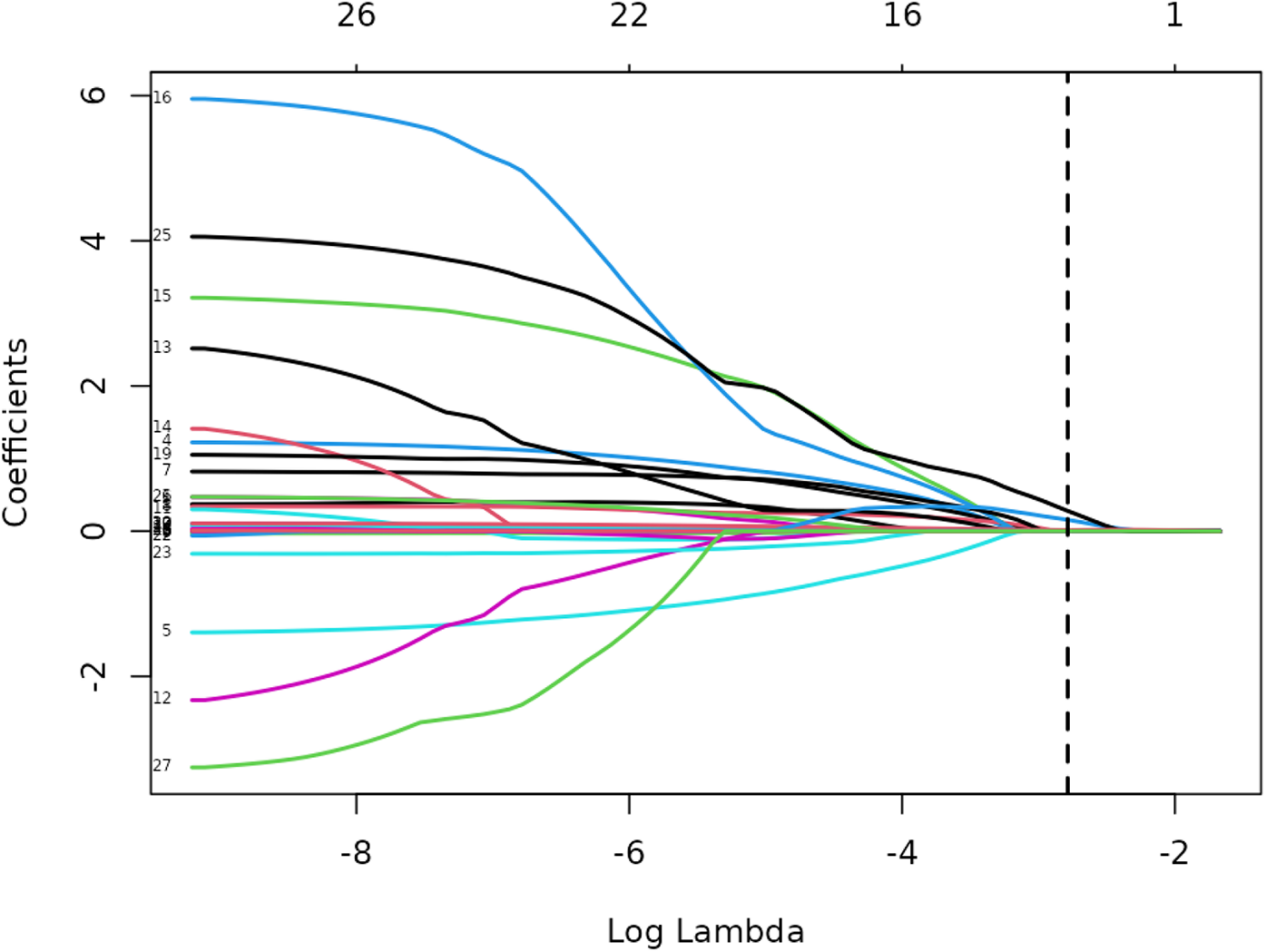
Path diagram of lasso regression variable selection.

Lasso regression analysis was performed on all independent variables with ten-fold cross-validation to screen the most representative predictors of vulnerable plaques, and the results are shown in Figures 3, 4. The location of the log(*λ*) parameter in this study was chosen to be the dashed line leaning to the left in Figure 3, i.e., the value of *λ* at which the model error is the smallest. Then the solution paths of Lasso regression coefficients containing 27 independent variables are plotted according to the optimal *λ* values screened in Figure 3, as shown in Figure 4, and four independent variables are finally screened out.

The variation of the independent variables in Lasso regression with the value of *λ* is shown in Figure 3: Figure 3 shows the relationship between log(*λ*) and Lasso regression coefficients, with the vertical coordinate is the model regression coefficients, the lower horizontal coordinate being log(*λ*), and the upper horizontal coordinate being the number of non-zero coefficients independent variables in the model corresponding to different log(*λ*). As *λ* increases, the degree of compression of each independent variable coefficient estimate increases, and the independent variable coefficients that have less impact on the model prediction results are compressed to 0, and the number of independent variables gradually decreases; Figure 4 shows the relationship between log(*λ*) and the corresponding number of independent variables, with the vertical coordinate being the model mean square error, the lower horizontal coordinate being log(*λ*), and the upper horizontal coordinate being the non-zero coefficients in the model of the independent variables corresponding to the different log(*λ*) Number. The left dotted line indicates lambda.min, which is the value of *λ* when the model mean square error is at its minimum, at which time the number of variables in the model is four. The right dotted line indicates lambda.1se, which is the value of *λ* when the model mean square error is at one standard error, at which time the number of variables in the model is two. In this study, we chose *λ* = 0.0617 as the optimal model, and then the independent variables screened by the Lasso regression were: IL-6, TSH, TSHI, and TT4RI, with corresponding beta values of −0.01, −0.16, −0.28, and −0.01, respectively.

#### 3.3.3 Vulnerable plaque risk factor analysis results and model evaluation

In this study, Lasso-logistic regression was performed and compared with all-variable logistic regression as well as stepwise logistic regression, and the results are shown in Table 3. IL-6 showed statistically significant (*P* < 0.05) in all of three models. Among them, the Lasso-logistic regression model AIC = 161.6376 and BIC = 176.0136 were lower than the all-variable logistic regression (AIC = 181.0881 BIC = 261.5936), and the BIC was smaller than the stepwise logistic regression (AIC = 154.024 BIC = 179.9007), suggesting that the Lasso-logistic regression model has a better model fitting performance.

**Table 3:** Comparison of three models for factors influencing the vulnerable carotid plaque.

Features	All-variable logistic regression model		Stepwise logistic regression model		Lasso-logistic regression model	
	*P*	OR (95%CI)	*P*	OR (95%CI)	*P*	OR (95%CI)
Gender (*n*, %)						
Female						
Male						
Age (years)	0.441	1.02 (0.97–1.07)				
BMI (kg/m^2^)						
Smoking history (*n*, %)						
No						
Yes			0.014	3.83 (1.32–11.13)		
Alcohol history (*n*, %)						
No						
Yes	0.377	0.62 (0.22–1.79)	0.023	0.218 (0.06–0.81)		
Hypertension (*n*, %)						
No						
Yes						
Type 2 diabetes (*n*, %)						
No						
Yes						
Stroke (*n*, %)						
No						
Yes	0.259	1.59 (0.71–3.54)				
Alb (g/L)						
Glu (mmol/L)						
TG (mmol/L)						
TC (mmol/L)			0.090	0.22 (0.04–1.26)		
HDL (mmol/L)			0.015	20.15 (1.81–224.86)		
LDL (mmol/L)						
Apo AⅠ (g/L)						
Apo B (g/L)			0.053	1,658.12 (0.91–3,010,764.95)		
CRP (mg/L)	0.308	1.06 (0.95–1.17)	0.267	1.06 (0.95–1.18)		
Hb (g/L)						
PCT (ng/ml)						
IL−6 (pg/ml)	0.015	1.05 (1.01–1.10)	0.004	1.07 (1.02–1.11)	0.008	1.05 (1.01–1.10)
D-dimer (mg/L)						
TSH (mIU/L)	0.758	1.33 (0.21–8.30)			0.287	2.04 (0.55–7.62)
FT3 (pmol/L)						
FT4 (pmol/L)						
TSHI	0.167	7.92 (0.42–148.70)	<.001	6.83 (2.78–16.80)	0.213	5.60 (0.37–84.26)
TT4RI	0.893	0.99 (0.83–1.18)			0.606	0.96 (0.84–1.11)
TFQI	0.590	0.38 (0.01–12.76)				
AIC	166.10		154.02		161.64	
BIC	194.85		179.90		176.01	

In addition, based on the four independent variables screened by Lasso, several prediction models were fitted with carotid plaque stability (stable plaque group or vulnerable plaque group) as the dependent variable, as shown in Supplementary Figure S2. Prediction models with favorable differentiation were screened based on the AUC > 0.75, yielding model 6, 7, 12, 13, and 15, and IL6 + TSHI was ultimately selected to construct the clinical prediction model. Reason: Model 6 includes only two independent variables, namely, variables IL6 and TSHI, relative to other models, so the model structure is relatively simple, easier to interpret and understand, and easier to apply in clinical practice. Fewer variables reduced the complexity of data collection and processing, as well as the risk of potential multicollinearity and overfitting in the models. According to above reasons, model 6 was finally selected to construct the clinical prediction model. The optimal threshold of the model is 0.46, with a specificity of 0.72 and a sensitivity of 0.85 (Supplementary Table S5).

**Figure 5:**
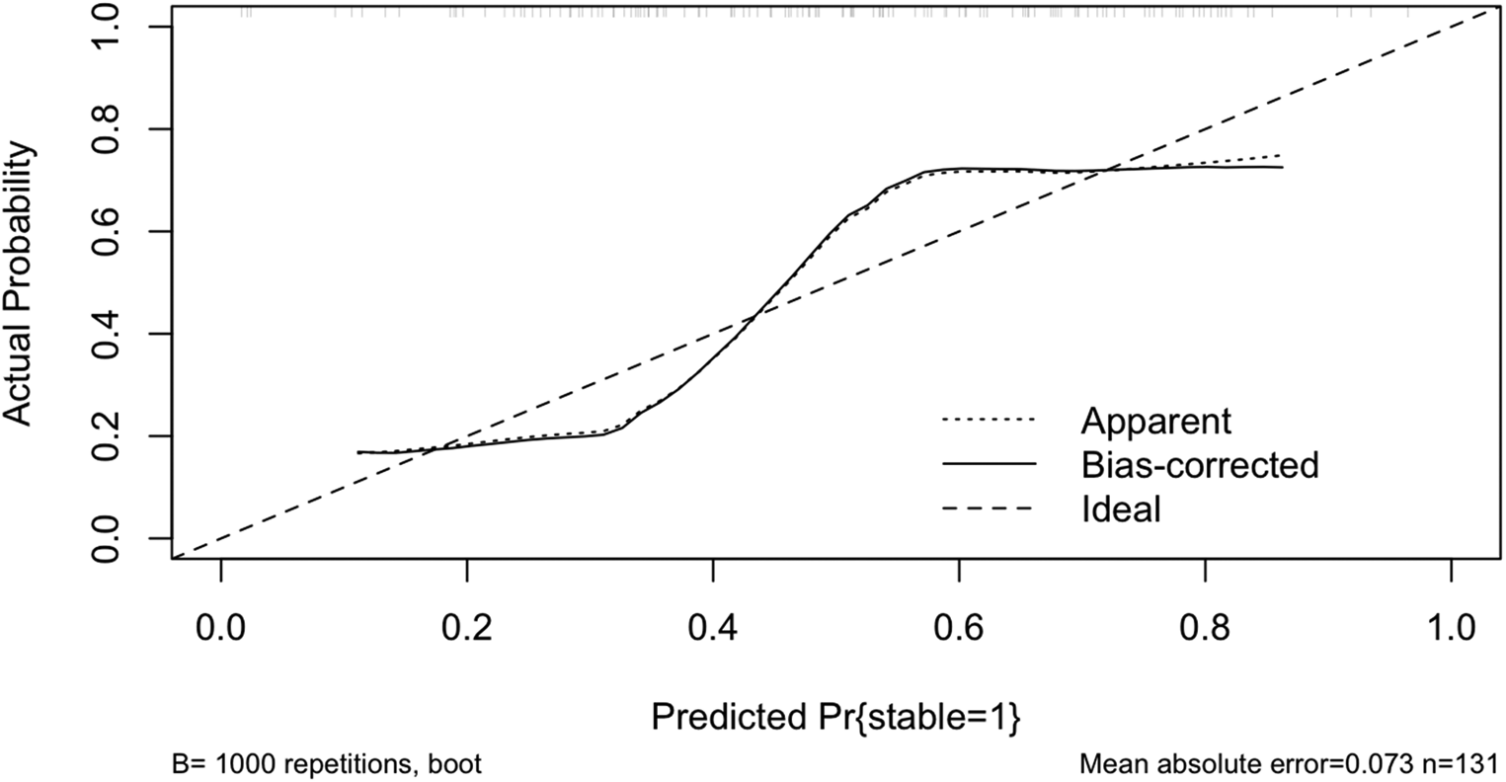
Calibration curve of lasso-logistic regression prediction model.

Figure 5 shows the calibration curve of the Lasso-logistic prediction model, where the horizontal coordinate indicates the observed probability and the vertical coordinate indicates the predicted probability, the long dashed line in the figure indicates the ideal state, the short dashed line Apparent is the risk probability based on the calculation of this model once in agreement with the actual probability, and the solid line Bias-corrected refers to the data of the constructed model for the self-lifting weight sampling Post-calibration curves. Bootstrap resampling refers to repeatedly (the number of times is usually 100 or 1,000) randomly selecting a sample of *n* observations from the original data evaluating the calibration of each sample, and then calculating the average value. Compared with calculating the risk probability at one time, this average risk probability based on a random sample better reflects whether the model is stable or not, and also avoids data with model overfitting. Mean Absolute Error (MAE) of the calibration curve is 0.073.

**Figure 6:**
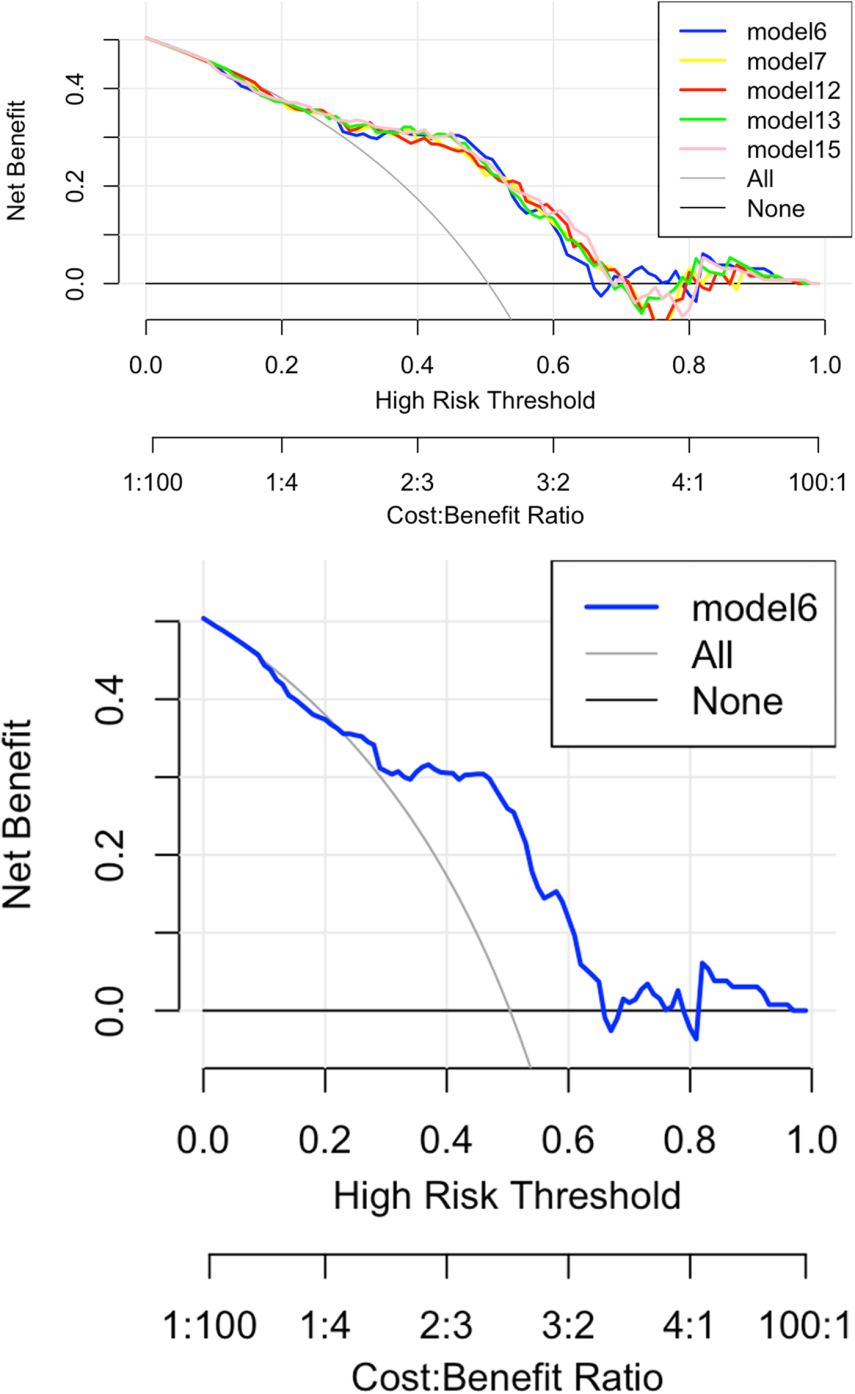
Decision curve analysis of lasso-logistic regression prediction model.

Figure 6 shows the decision curve analysis (DCA) of the Lasso-logistic prediction model. The final DCA curve showed that if the threshold probability of patients or clinicians is between 25% and 65%, using this model based on our nomogram to predict vulnerable plaque adds more benefit than either screen-none or screen-all strategies.

**Figure 7:**
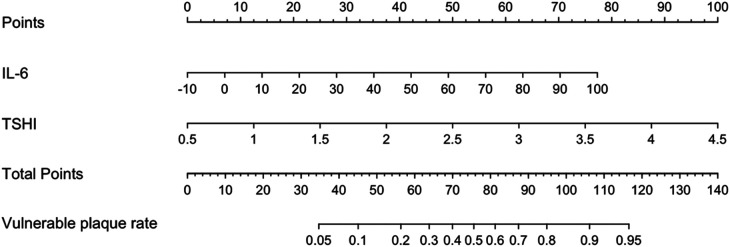
Nomogram of lasso-logistic regression prediction model.

Figure 7 shows a nomogram of the Lasso-logistic regression prediction model, visualizing the predicted score of vulnerable carotid plaques. For example, a carotid artery stenosis patient with an IL-6 level of 20 pg/ml and a TSHI level of 3 corresponding to a total score of 100 has a probability predictive value of approximately 0.85 for vulnerable carotid plaques.

## 4 Discussion

The occurrence of vulnerable plaques in patients with carotid artery stenosis is influenced by a combination of different factors. In this study, we explored the correlation between carotid plaque stability and a series of clinical factors, ultimately found that IL-6 as well as the TSHI had a certain predictive efficacy for carotid plaque stability. The study aimed to compare the disease characteristics of stable and vulnerable plaque populations in a single-center retrospective study, to find the independent risk factors of vulnerable carotid plaque. Risk factors would be used to establish a noninvasive diagnostic model for vulnerable plaque based on Lasso-logistic regression. This model would provide a new reference tool for the clinical diagnosis of vulnerable plaque.

The results of this study suggest that IL-6 is an independent risk factor for vulnerable carotid plaque, which is consistent with previous correlative studies in coronary arteries (31). Similar studies have also shown that inflammatory markers such as IL-6, TNF-α, and C-reactive protein can predict vulnerable carotid plaque (32). Specific mechanisms may be activation of inflammation, vascular endothelial dysfunction, oxidative stress, Th17 cell differentiation, JAK/STAT pathway activation, and altered lipid metabolism. Specifically, IL-6 is secreted as a circulating cytokine in a wide range of cells (including macrophages, monocytes, fibroblasts, and endothelial cells) and serves as a potent inducer and proinflammatory factor for Th17 cells.IL-6 signaling induces a downstream inflammatory response leading to an elevation of acute-phase reactants, such as high-sensitivity C-reactive protein, fibrinogen, etc., and therefore contributes to an increase in atherosclerosis (33). In addition, IL-6 activates chronic inflammation through the JAK/STAT pathway and increases the expression of adhesion molecules in the vasculature, leading to endothelial dysfunction, monocyte/macrophage recruitment and smooth muscle cell migration. These processes lead to increased lipid deposition, plaque development, and plaque instability, and IL-6 exacerbates the atherosclerotic process by up-regulating RUNX2 and RANKL/RANK gene expression, which promotes the differentiation of vascular smooth muscle cells to osteoblasts, which in turn causes the deposition of calcium-phosphorus complexes (34).

Thyroid hormone sensitivity indexes in this study including TSHI, TT4RI, and TFQI are used to assess the sensitivity of thyroid hormones to feedback regulation of the hypothalamic-pituitary-thyroid axis. They are more stable than the single indexes such as TSH, FT3, and FT4. The results of univariate logistic regression analysis in this study showed that the vulnerable plaque group had higher levels of TSHI, TT4RI, and TFQI, suggesting that reduced central sensitivity to thyroid hormones is associated with vulnerable plaques. The interaction between inflammation and thyroid hormone sensitivity plays a critical role in calcium and phosphorus metabolism, which may contribute to atherosclerosis development. Inflammatory cytokines like IL-6 can disrupt thyroid hormone activity, altering calcium homeostasis by affecting bone resorption and vascular calcification (35). In our study, four independent variables, IL-6, TSH, TSHI, and TT4RI, were finally screened based on Lasso regression, this further supports the aforementioned viewpoint from another perspective. Although the statistical significance of the thyroid-related sensitivity indices was not less than 0.05, they were still retained after screening by Lasso regression, because statistical significance (e.g., *p*-value) and clinical relevance were not always consistent and they were considered to contribute to the final model. The correlation indexes were also suggested to be associated with the final outcome in univariate logistic regression. Based on the conclusions of previous studies, this study finally chose IL-6 and TSHI as predictors to construct a simple clinical prediction model of vulnerable carotid plaque. Related studies have shown that thyroid dysfunction can have profound effects on cardiac metabolism and hemodynamics, with both subclinical hyperthyroidism and hypothyroidism associated with increased systolic and diastolic blood pressure, and thus increased cardiovascular risk (36). A more evidence-graded study using a Mendelian randomization methodology approach to assess the relationship between thyroid function and atherosclerosis found that lower free thyroxine (FT4) levels in the normal range were significantly associated with increased carotid intima-media thickness (CIMT). This study suggests that there may be a U-shaped relationship between FT4 levels and CIMT, suggesting that thyroid hormone supplementation in hypothyroid patients may help to reduce CIMT, thereby reducing the risk of carotid atherosclerosis. This study emphasizes the importance of monitoring thyroid function for cardiovascular risk assessment and implies that thyroid hormone supplementation therapy may play a positive role in mitigating atherosclerosis risk (37). Our study found that reduced thyroid hormone sensitivity, particularly central resistance, may exacerbate progress of atherosclerosis by linking metabolic dysregulation to plaque vulnerability. Previous studies have shown that gender factors and hypertension are also related to carotid plaque stability (38), while gender and hypertension were not the influencing factors of plaque stability in this study, and we hypothesized that it might be related to the small sample size included in this study.

Alcohol consumption was found to be significantly different between the stable and vulnerable plaque groups in the baseline comparison (*p* = 0.047). However, this association did not reach statistical significance in the univariate logistic regression analysis (*p* = 0.051). After adjusting for other variables in multivariate analysis, alcohol consumption demonstrated a different effect. This discrepancy highlights the influence of different statistical methods, as the chi-square test assesses group differences while logistic regression evaluates the independent effect of variables. Additionally, the relatively small sample size may have limited the statistical power of the logistic regression, leading to borderline significance. Moreover, alcohol consumption's potential impact on the hypothalamic-pituitary-thyroid axis, as previously reported, may contribute to the observed association between TSHI and plaque vulnerability (39). These findings emphasize the need for larger cohort studies to further explore the role of alcohol consumption in carotid plaque stability.

Several recent studies have investigated serum markers to predict carotid plaque vulnerability, identifying specific biomarkers that show promise for clinical application. For instance, a study examined the use of MMP-9, LOX-1, and YKL-40. It found that the combined use of these three markers achieved a high diagnostic accuracy (ROC AUC = 0.85) for identifying vulnerable plaques (16). Another study demonstrated that combining miR-124, IL-1β, and TNF-α achieved an AUC of 0.85, with a sensitivity of 0.83 and specificity of 0.79 (40). Our model using IL-6 and TSHI achieved an AUC of 0.77, accuracy of 0.79, sensitivity of 0.85, and specificity of 0.72. Although our model's AUC is slightly lower, but its high sensitivity demonstrates potential for identifying vulnerable plaques in clinical applications.

In this study, we developed a sample to operate and visualize column-line graph prediction model based on the Lasso-logistic regression model. The high predictive efficacy of the column-line graph model was confirmed by ROC curves. It is expected that the Lasso-logistic regression-based column-line diagram model developed in this study has a high clinical potential for predicting vulnerable carotid plaques. Of course, this study has some limitations. Firstly, this study is a retrospective study, and the patients all suffered from carotid artery stenosis and underwent carotid endarterectomy, which may have some selective bias. Secondly, despite the rigorous control for common comorbidities such as hypertension, diabetes, and stroke in our study, the retrospective nature of our study posed certain limitations. Specifically, other less common comorbid conditions and medication use (antiplatelet and lipid-lowering therapies) were not accounted for, which may have introduced unmeasured confounding factors. Future studies should consider a broader range of comorbidities and medication exposures to enhance the robustness of the findings. Once again, this study was a single-center investigation with a small sample size and a limited number of female participants, which may have introduced gender bias and affected the statistical significance of the results. Larger, prospective studies are needed in the future to achieve gender balance, provide more comprehensive insights into the studied population, and validate the findings.

Histologic classification is the gold standard for evaluating atherosclerotic plaques (41), so this study took advantage of the ability to obtain a certain amount of carotid plaque specimens and used a pathologic method to assess plaque stability. In order to give more accurate results of carotid plaque stability compared with imaging methods. However, considering that stable and vulnerable plaques were only based on pathological sections and immunohistochemical results, there was a subjective nature of manual scoring, and quantitative analysis was not used. Subsequent studies would be better to use quantitative analysis to accurately assess plaque stability through more specific metrics such as, for example, intraplaque hemorrhage, inflammation, and lipid necrotic cores.

In conclusion, the present study utilized IL-6 and TSHI to establish a column-line graph prediction model based on Lasso-logistic regression as a quantitative tool for the clinical diagnosis of vulnerable plaques, which has high diagnostic efficacy and benefit, and is worthy of promotion and application.

## Data Availability

The original contributions presented in the study are included in the article/Supplementary Material, further inquiries can be directed to the corresponding author.
