# Supplementary material for: Interleukin-6 and thyroid-stimulating hormone index predict plaque stability in carotid artery stenosis: analyses by lasso-logistic regression
Source: Front Cardiovasc Med. 2024 Dec 9;11:1484273. doi: 10.3389/fcvm.2024.1484273 (PMC11663930; doi:10.3389/fcvm.2024.1484273)
Supplement: Supplementary file 1 [file Image1.pdf]

# Supplementary Figures

## Supplementary Figure 1: Histological images and plaque vulnerability scores

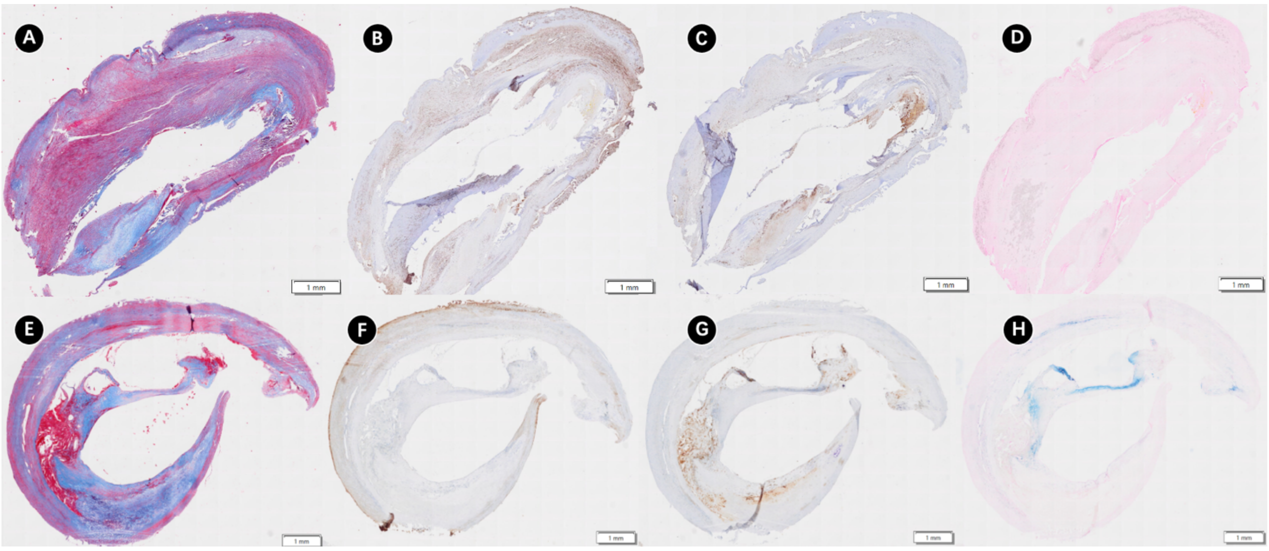


Note: The scale bars in all immunohistochemical images represent 1 mm.

A vulnerable plaque **(A–D)**, with a total score of 4 points.

**(A)** Masson staining shows a low collagen fiber content within the plaque (score: 1).

**(B)** α-SMA immunohistochemistry shows a low content of contractile smooth muscle cells within the plaque (score: 1).

**(C)** CD68 immunohistochemistry shows significant macrophage infiltration within the plaque (score: 1).

**(D)** Prussian blue staining shows evidence of hemorrhage within the plaque (score: 1).

A stable plaque **(E–H)**, with a total score of 1 point.

**(E)** Masson staining shows a high collagen fiber content within the plaque (score: 0).

**(F)** α-SMA immunohistochemistry shows a low content of contractile smooth muscle cells within the plaque (score: 1).

**(G)** CD68 immunohistochemistry shows minimal macrophage infiltration within the plaque (score: 0).

**(H)** Prussian blue staining shows no evidence of hemorrhage within the plaque (score: 0).

## Supplementary Figure 2: ROC curves of different prediction models

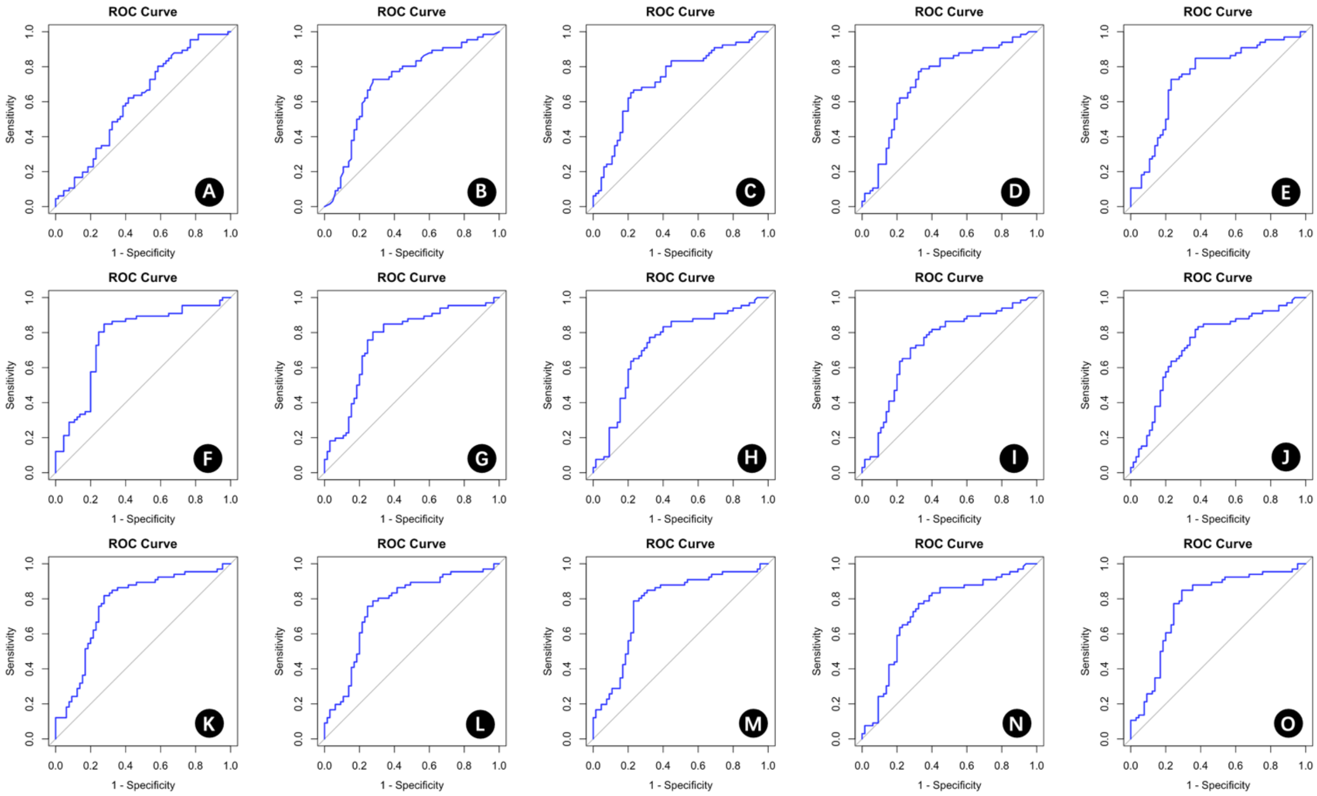


Note: ROC curves (A–O) represent the predictive performance for Models 1–15, respectively. Each panel (A–O) corresponds to one model, with sensitivity on the y-axis and 1-specificity on the x-axis.
