# Supplementary material for: Interleukin-6 and thyroid-stimulating hormone index predict plaque stability in carotid artery stenosis: analyses by lasso-logistic regression
Source: Front Cardiovasc Med. 2024 Dec 9;11:1484273. doi: 10.3389/fcvm.2024.1484273 (PMC11663930; doi:10.3389/fcvm.2024.1484273)
Supplement: Supplementary file 2 [file Table1.pdf]

Supplementary Tables

Supplementary Table 1: List of abbreviations

Abbreviations	Full description
AIC	Akaike information criterion
Alb	Albumin
Apo AI	Apolipoprotein AI
Apo B	Apolipoprotein B
AUC	Area Under the Curve
BIC	Bayesian information criterion
BMI	Body Mass Index
CIMT	intima-media thickness
CRP	C-Reactive Protein
Glu	Glucose
Hb	Hemoglobin
HDL	High-Density Lipoprotein Cholesterol
IL-6	Interleukin-6
LDL	Low-Density Lipoprotein Cholesterol
PCT	Procalcitonin
ROC	Receiver Operating Characteristic
TC	Total Cholesterol
TFQI	Thyroid Feedback Quantile-based Index
TG	Triglycerides
TSH	Thyroid-Stimulating Hormone
TSHI	Thyroid-Stimulating Hormone Index
TT4RI	Thyrotroph T4 Resistance Index

Supplementary Table 2: Calculation of Thyroid Sensitivity Indices

TSHI	TSH index	TSHI=ln TSH+0.1345*FT4
TT4RI	Thyrotroph T4 Resistance Index	TT4RI=FT4*TSH
TFQI	Thyroid Feedback Quantile-based Index	TFQI=cdf FT4-（1-cdf TSH）

Note: cdf represents the cumulative distribution function.

Supplementary Table 3: Vulnerable Plaque Score Data Sheet for Both Groups of Patients

Total Scores	Total Cases（n=131）
0	11
1	21
2	33
3	37
4	29
Stable plaques（Scores≤2）	65

Vulnerable plaques （Scores>2）	66

Supplementary Table 4: Results of covariance diagnosis

Features	Variance Inflation Factor (VIF)	Tolerance
Gender	1.63	0.61
Age	1.63	0.61
BMI	1.96	0.51
Smoking history	1.94	0.52
Alcohol history	2.04	0.49
Hypertension	1.48	0.67
Type 2 diabetes	1.84	0.54
Stroke	1.33	0.75
Alb	1.64	0.61
Glu	1.54	0.65
TG	8.63	0.12
**TC**	**128.05**	**0.01**
**HDL**	**12.11**	**0.08**
**LDL**	**107.77**	**0.01**
Apo AI	4.24	0.24
**Apo B**	**27.70**	**0.04**
CRP	1.49	0.67
Hb	1.83	0.55
PCT	1.62	0.62
IL-6	2.59	0.39
D-dimer	1.60	0.63
**TSH**	**67.68**	**0.01**
FT3	2.00	0.50
**FT4**	**30.17**	**0.03**
**TSHI**	**20.08**	**0.05**
**TT4RI**	**59.99**	**0.02**
**TFQI**	**41.24**	**0.02**

Supplementary Table 5: Performance of different models based on Lasso-logistic regression

Model	AUC	P	95%CI	Threshold	Accuracy	Sensitivity	Specificity
Model1:IL6	0.61	0.026	0.52-0.71	0.45	0.61	0.80	0.42
Model2:TSH	0.72	<.001	0.62-0.81	0.47	0.73	0.73	0.72
Model3:TSHI	0.73	<.001	0.64-0.82	0.54	0.72	0.65	0.78
Model4:TT4RI	0.73	<.001	0.64-0.82	0.45	0.73	0.77	0.68
Model5:IL6+TSH	0.74	<.001	0.66-0.83	0.48	0.75	0.73	0.77
**Model6:IL6+TSHI**	**0.77**	**<.001**	**0.68-0.85**	**0.46**	**0.79**	**0.85**	**0.72**
Model7:IL6+TT4RI	0.76	<.001	0.67-0.84	0.42	0.76	0.80	0.72

Model8:TSH+TSHI	0.73	<.001	0.64-0.82	0.47	0.73	0.77	0.68
Model9：TSH+TT4RI	0.73	<.001	0.64-0.82	0.47	0.72	0.71	0.72
Model10：TSHI+TT4RI	0.73	<.001	0.64-0.82	0.43	0.73	0.82	0.63
Model11：IL6+TSH+TSHI	0.77	<.001	0.68-0.85	0.45	0.77	0.82	0.72
Model12：IL6+TSH+TT4RI	0.76	<.001	0.67-0.84	0.47	0.76	0.76	0.75
Model13：IL6+TSHI+TT4RI	0.77	<.001	0.69-0.85	0.46	0.78	0.79	0.77
Model14：TSH+TSHI+TT4RI	0.73	<.001	0.64-0.82	0.47	0.73	0.77	0.68
Model15：IL6+TSH+TSHI+TT4RI	0.77	<.001	0.69-0.85	0.45	0.78	0.85	0.71
